# Apelin‐13 Attenuates Blood–Brain Barrier Dysfunction Following Intracerebral Hemorrhage via Targeting the Keap1/Nrf2 Signaling

**DOI:** 10.1002/cns.70706

**Published:** 2025-12-29

**Authors:** Pingping Guo, Rabeea Siddique, Juanfeng Qian, Lingxiao Qi, V. Wee Yong, Mengzhou Xue

**Affiliations:** ^1^ Department of Cerebrovascular Diseases The Second Affiliated Hospital of Zhengzhou University Zhengzhou Henan China; ^2^ Academy of Medical Science, Zhengzhou University Zhengzhou Henan China; ^3^ Hotchkiss Brain Institute and Department of Clinical Neurosciences University of Calgary Calgary Alberta Canada

**Keywords:** Apelin‐13, blood–brain barrier, intracerebral hemorrhage, Keap1/Nrf2, oxidative stress

## Abstract

**Background:**

Blood–brain barrier (BBB) dysfunction serves as a critical driver of the secondary brain injury following intracerebral hemorrhage (ICH). Previous research has indicated that Apelin‐13 demonstrates the potential to alleviate BBB dysfunction in various cerebrovascular disorders. However, the precise mechanisms through which Apelin‐13 preserves BBB integrity remain elusive. This study investigated whether Apelin‐13 exerted neuroprotective effects by targeting the Keap1/Nrf2 signaling.

**Methods:**

An in vivo ICH model was established using collagenase. Neurological function, brain edema, tight junction protein levels, and Evans blue leakage were assessed. In vitro, bEnd.3 monolayers were induced by hemin to simulate ICH conditions. To assess the role of Apelin‐13 in the Keap1/Nrf2 signaling, we employed specific shRNAs targeting Nrf2 and apelin receptor (APJ). The neuroprotective effects of Apelin‐13 in hemin‐stimulated bEnd.3 cells were assessed through transendothelial electrical resistance assay, western blotting, and immunofluorescence analysis.

**Results:**

Apelin‐13 treatment significantly mitigated brain damage, reduced cerebral edema, and promoted neurological recovery in ICH mice. These effects were accompanied by a significant decrease in matrix metalloproteinase‐9 expression and an increase in tight junction protein levels. Similar protective effects were observed in hemin‐induced bEnd.3 cells, where Apelin‐13 additionally promoted Nrf2 expression and suppressed Keap1 expression, suggesting the involvement of the Keap1/Nrf2 signaling. Critically, APJ silencing blocked the effects of Apelin‐13 on the Keap1/Nrf2 pathway. Furthermore, Nrf2 knockdown eliminated the protective effects of Apelin‐13, reversing its attenuation of apoptosis, preservation of tight junction integrity, and reduction of oxidative stress in hemin‐stimulated bEnd.3 cells.

**Conclusions:**

In conclusion, these findings demonstrate that through its engagement with APJ, Apelin‐13 activates the endothelial Keap1/Nrf2 pathway to protect against ICH‐induced BBB disruption and facilitate neurological recovery, highlighting its therapeutic promise for ICH.

## Graphical abstract

Apelin‐13 attenuates BBB disruption following intracerebral hemorrhage both in vivo and in vitro. Mechanistically, Apelin‐13 activates the endothelial Keap1/Nrf2 signaling pathway and enhances its antioxidant defenses, in which APJ is a critical mediator.

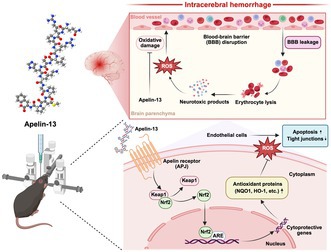

## 1 Introduction

As a devastating stroke subtype, intracerebral hemorrhage (ICH) is responsible for 50% of stroke‐related mortality and often causes long‐term neurological disability in survivors [1]. Following ICH, a deleterious cascade of secondary brain injury is triggered, in which blood–brain barrier (BBB) compromise and oxidative stress play critical roles [2]. BBB dysfunction is a key driver of perihematomal brain edema, leading to intracranial hypertension and poor prognosis. Specifically, BBB breakdown mediates vasogenic edema by enabling a hydrostatic pressure‐driven influx of fluid, which is most evident in white matter [3]. Furthermore, extravasated red blood cells from the compromised BBB release hemoglobin, iron, and heme. These cytotoxic components drive the overproduction of reactive oxygen and nitrogen species (ROS/RNS), overwhelming endogenous antioxidant capacity and resulting in oxidative damage to cellular elements like lipids, proteins, and DNA [4]. The accumulation of these cytotoxic products not only induces cellular dysfunction but also elevates tissue osmolality, thereby triggering cytotoxic edema that is primarily observed in gray matter [5]. Concurrently, the resulting oxidative stress further exacerbates BBB disruption by damaging endothelial cells and degrading extracellular matrix and tight junction proteins [6, 7]. Therefore, therapeutic interventions that counteract oxidative stress hold significant potential for alleviating BBB impairment and brain edema following ICH.

Nuclear factor erythroid 2‐related factor 2 (Nrf2) functions as a crucial regulator to orchestrate the transcription of cytoprotective genes [8]. During cellular homeostasis, Kelch‐like ECH‐associated protein 1 (Keap1) anchors Nrf2 in the cytoplasm, facilitating its ubiquitination and proteasomal degradation [9]. Nevertheless, when exposed to ROS/RNS, Keap1 undergoes modifications that impair Nrf2 ubiquitination, contributing to Nrf2 nuclear accumulation and subsequent cytoprotective gene activation [10]. Evidence demonstrates that Nrf2 transcriptional inhibition disrupts redox homeostasis and metabolic processes in endothelial cells, concomitant with downregulation of tight junction protein expression, ultimately compromising BBB integrity [11]. Conversely, Nrf2 activation confers neuroprotection against brain injury in an ischemic stroke model by attenuating BBB disruption and reducing brain edema through enhanced antioxidant defenses [12, 13]. Taken together, Nrf2 signaling constitutes a promising therapeutic target to mitigate ICH‐induced BBB dysfunction and alleviate secondary brain injury. A growing body of evidence highlights the therapeutic potential of various Nrf2 activators for ICH, with promising candidates including sulforaphane, flavonoids, fumarates, curcumin, 5α‐androst‐3β,5α,6β‐triol (TRIOL), baicalin, and so on [14, 15, 16]. Despite their demonstrated neuroprotective effects, no Nrf2‐targeting agents have yet been translated into clinical practice for ICH management. Thus, it is critically urgent to identify novel Nrf2‐targeting neuroprotective agents and achieve their clinical application in ICH therapy.

Apelin is an endogenous ligand for the APJ receptor, which belongs to the G protein‐coupled receptor family. The Apelin/APJ system is widely distributed throughout the central nervous system, with particularly high abundance in endothelial cells, highlighting its potential importance in regulating endothelial function [17, 18]. Among all apelin isoforms, Apelin‐13 demonstrates the highest biological activity [19]. Accumulating evidence has indicated that Apelin‐13 exerts neuroprotective effects in multiple cerebrovascular pathologies. In subarachnoid hemorrhage models, Apelin‐13 dose‐dependently mitigated BBB disruption and reduced cerebral edema by suppressing oxidative stress and neuroinflammation [20]. In ischemic stroke models, it has been shown to alleviate neuroinflammation, attenuate BBB breakdown, regulate oxidative stress, and inhibit pyroptosis [21, 22, 23]. Additionally, emerging evidence suggests that Apelin‐13 also confers neuroprotection in ICH through alleviating BBB dysfunction and suppressing cell apoptosis [24]. Given these multifaceted neuroprotective effects demonstrated in various cerebrovascular disease models, Apelin‐13 represents a highly promising candidate for ICH treatment. Mechanistically, previous studies indicated that Apelin‐13 exerted protective effects in ischemic stroke and Alzheimer's Disease through activating Nrf2 signaling [25, 26]. However, the precise molecular mechanisms through which Apelin‐13 protects against brain injury following ICH remain to be fully elucidated.

This study utilized in vivo and in vitro ICH models to systematically investigate Apelin‐13's effectiveness in preserving BBB integrity and mechanistically dissect its engagement with the Keap1/Nrf2 signaling.

## 2 Materials and Methods

### 2.1 Animals

Male C57BL/6 mice aged 6–8 weeks were sourced from Beijing Vital River Laboratories. All animals were maintained under specific pathogen‐free conditions with controlled temperature, a 12‐h light/dark cycle, and free access to food and water. All animal experiments adhered to the National Animal Welfare Guidelines of China (GB/T 35892‐2018) and received approval from the Ethics Committee of the Second Affiliated Hospital of Zhengzhou University (Approval No. KY2024231).

### 2.2 ICH Model

The ICH model was induced according to a previous study [27]. Following anesthesia via intraperitoneal injection of a ketamine (100 mg/kg) and xylazine (10 mg/kg) mixture, a solution of collagenase VII (0.075 U in 0.75 μL saline; Sigma, USA) was infused into the right striatum. The sham group received an equivalent volume of sterile PBS. Apelin‐13 (60 μg/kg; Santa Cruz, USA) was administered intracerebroventricularly immediately after ICH induction followed by daily injections, as previously reported [25]. Concurrently, the vehicle control group received intracerebroventricular injections of the equivalent volume of sterile PBS. After the surgical procedure, mice were placed in a warm environment and closely monitored until complete recovery from anesthesia.

### 2.3 Behavioral Tests

Neurological function was evaluated at baseline, Day 1, and Day 3 following ICH induction using the forelimb placing test and rota‐rod test, as previously described [28, 29].

### 2.4 Hematoxylin and Eosin (H&E) Staining

Whole brains were harvested at Day 3 post‐ICH following transcardial perfusion with PBS and 4% paraformaldehyde. The brain tissue sections were routinely processed for histological examination. Following deparaffinization and rehydration, sections were stained with hematoxylin solution for 5 min to visualize nuclear details. This was followed by differentiation in rapid differentiation solution and a rinse in running tap water. Subsequently, sections were incubated with eosin solution for 2 min to delineate cytoplasmic and extracellular matrix components. After dehydration, the slides were coverslipped with a mounting medium. Finally, sections were imaged on a slide scanner (G Cell, China).

### 2.5 Brain Water Content Assessment

Whole brains were collected and dissected into five distinct regions: ipsilateral cortex, contralateral cortex, ipsilateral basal ganglia, contralateral basal ganglia, and cerebellum. Each dissected brain region was initially weighted to record the wet weight (WW), followed by oven‐drying at 110°C for 24 h until completely dehydrated, before a final weighing to record the dry weight (DW). Results were presented according to the formula: (WW–DW)/WW × 100% [30].

### 2.6 Evans Blue (EB) Assay

A 2% EB (4 mL/kg; Sigma) solution was injected intravenously through the tail vein. Three hours after injection, the mice underwent transcardial perfusion with PBS to eliminate intravascular dye. Whole brain was collected and separated into left and right hemispheres. This was followed by homogenization with formamide and incubation at 60°C for 48 h. After centrifugation, the supernatants were collected, and its absorbance was measured at 610 nm with a SpectraMax M5/M5e spectrophotometer (Molecular Devices, USA). The EB concentration was obtained from a standard curve and presented as the ipsilateral‐to‐contralateral ratio of EB concentration.

### 2.7 Cell Culture

The bEnd.3 mouse brain endothelial cells (Procell, China) were cultured on 12 mm diameter transwell inserts with a 0.4 μm pore size (Corning Life Sciences, USA) for 48 h to establish confluent monolayers. Hemin (80 μM; Sigma) was used to induce in vitro ICH models. Prior to hemin exposure, cells were pretreated overnight with Apelin‐13 at concentrations of 0.5, 1, and 2 μM.

### 2.8 Cell Viability Assay

Following treatment with hemin (0–200 μM) or Apelin‐13 (0–50 μM) for 24 h, cell viability was assessed with the Cell Counting Kit‐8 (CCK‐8) assay. Briefly, bEnd.3 cells were incubated with the CCK‐8 reagent (GlpBio, USA) at 37°C for 2 h, and the absorbance at 450 nm was measured. Three repeated tests were performed. Calcein‐AM/PI staining was performed following manufacturer's instructions to evaluate the live/dead cell ratio. Images acquisition was performed on a Leica STELLARIS 5 SR laser confocal microscope (Leica, Germany).

### 2.9 Cell Transfection

Short hairpin RNAs (shRNAs) targeting Nrf2 and APJ were obtained from Santa Cruz Biotechnology. Cell transfection was performed according to the manufacturer's protocol. A control shRNA plasmid (Santa Cruz) was used as the negative control. Culture the transfected cells in the fresh medium for 48 h to achieve efficient knockdown.

### 2.10 Western Blot Analysis

Total protein was isolated from the perihematomal brain tissues or cultured bEnd.3 cells. Nuclear and cytoplasmic proteins were fractionated from bEnd.3 cells with a commercial extraction kit (Beyotime). After SDS‐PAGE separation, proteins were transferred onto 0.45 μm PVDF membranes, which were subsequently blocked with a 5% solution of non‐fat powered milk. Following overnight incubation with primary antibodies at 4°C and thorough washing with TBST, membranes were incubated with appropriate secondary antibodies for 1 h at room temperature. Protein bands were identified on an Amersham imager 600 (GE Life Sciences, USA). Table 1 provides the detailed antibody information involved in this experiment.

**TABLE 1:** Antibody information for the study.

Antibody	Dilution ratio	Catalog number	Manufactures
Western blot analysis			
ZO‐1	1:5000	21,773–1‐AP	Proteintech
Occludin	1:1000	ab216327	Abcam
MMP‐9	1:1000	ab283575	Abcam
Bcl2	1:2000	ab182858	Abcam
Bax	1:1000	14796S	Cell Signaling
Cleaved caspase‐3	1:1000	MAB835	R&D systems
Nrf2	1:2000	16,396–1‐AP	Proteintech
Keap 1	1:2000	10,503–2‐AP	Proteintech
NQO1	1:1000	DF6437	Affinity
HO‐1	1:2000	ER1802‐73	Huabio
APJ	1:1000	sc‐517,300	Santa Cruz
β‐Actin	1:5000	AP0060	BioWorld
Histone H3	1:2000	17,168–1‐AP	Proteintech
Goat Anti‐Rabbit IgG (HRP)	1:5000	ab205718	Abcam
Goat Anti‐Mouse IgG (HRP)	1:5000	ab205719	Abcam
Immunofluorescence staining			
ZO‐1	1:100	ab221547	Abcam
CD31	1:20	AF3628	R&D systems
Nrf2	1:200	16,396–1‐AP	Proteintech
Dylight 488 Goat Anti‐Rabbit IgG	1:200	A23220	Abbkine
IFKine Red Donkey Anti‐Goat IgG	1:200	A24431	Abbkine

### 2.11 Transendothelial Electrical Resistance (TEER) Measurement

Cells were seeded onto transwell filters, and the TEER of bEnd.3 monolayers was determined with a Cell Resistance Meter RE1600 (Beijing Kingtech Technology, China). The TEER (Ω·cm^2^) was calculated as follows: [TEER sample (Ω)−TEER blank (Ω)] × 1.12 (cm^2^).

### 2.12 Immunofluorescence Staining

Brain sections and bEnd.3 cells were blocked using 5% bovine serum albumin for 1 h after permeabilization. Following this, the samples were incubated at 4°C overnight with primary antibodies, followed by incubation at room temperature for 1 h with either Dylight 488 Goat Anti‐Rabbit IgG or IFKine Red Donkey Anti‐Goat IgG. A confocal microscope (Leica, Germany) was used for imaging. Table 1 summarizes the antibodies used in this experiment.

### 2.13 ROS Measurement

To evaluate intracellular oxidative stress, ROS levels were assessed using a fluorescent probe‐based ROS Assay Kit (Jiancheng Technology, China). Cells were incubated with 10 μM DCFH‐DA solution for 30 min. Fluorescence intensity was quantified with a Feyond‐A400 multifunctional microplate reader (Aosheng Instrument, China) with excitation wavelength at 488 nm. A confocal microscope (Leica) was used to visualize ROS distribution.

### 2.14 Enzyme‐Linked Immunosorbent Assay (ELISA)

Supernatants from bEnd.3 cells after different treatments were collected. Matrix metalloproteinase‐9 (MMP‐9) levels were quantified with an ELISA kit (Multi Sciences, China).

### 2.15 Biochemical Analysis

The intracellular concentrations of malondialdehyde (MDA), superoxide dismutase (SOD), glutathione (GSH), glutathione peroxidase (GSH‐Px), and catalase (CAT) in bEnd.3 cells were quantified with commercially available kits obtained from Jiancheng Technology, according to the manufacturer's guidelines.

### 2.16 qRT‐PCR Analysis

Total RNA isolation from bEnd.3 cells was performed with a Total RNA Extraction Kit (BioFlux, China). cDNA was synthesized using a Reverse Transcription Kit (TaKaRa, Japan). Quantitative real‐time PCR (qRT‐PCR) was performed on a QuantStudio 5 system (Thermo Fisher Scientific, USA) using SYBR Green PCR Master Mix (TaKaRa). The 2^−ΔΔCT^ method was used to calculate relative mRNA expression levels. The experiment was conducted with three biological replicates. Table 2 describes the primer sequences used in this experiment.

**TABLE 2:** Primers sequences for qRT‐PCR.

Gene	Primer	Sequence
NQO1	Forward	AGGATGGGAGGTACTCGAATC
	Reverse	AGGCGTCCTTCCTTATATGCTA
HO‐1	Forward	AAGCCGAGAATGCTGAGTTCA
	Reverse	GCCGTGTAGATATGGTACAAGGA
β‐Actin	Forward	GGCTGTATTCCCCTCCATCG
	Reverse	CCAGTTGGTAACAATGCCATGT

### 2.17 Statistical Analysis

All data were presented as mean ± standard deviation (SD). Statistical analyses were performed using one/two‐way ANOVA followed by Tukey's multiple comparison test in GraphPad Prism (Version 10.1.1). *p* < 0.05 was considered statistically significant.

## 3 Results

### 3.1 Apelin‐13 Ameliorated Brain Edema and Neurological Deficits in ICH Mice

**FIGURE 1:**
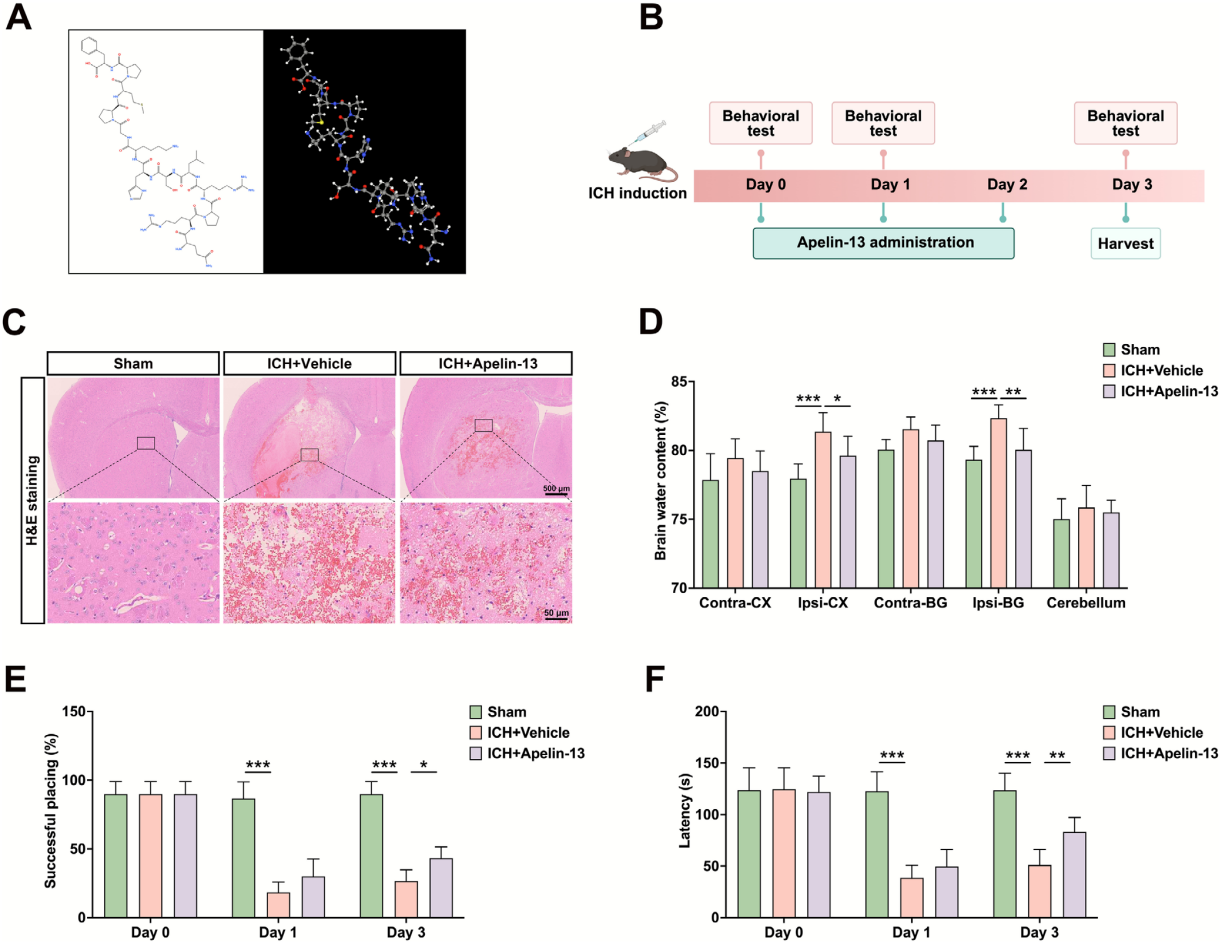
Apelin‐13 mitigated brain edema and enhanced neurological recovery in ICH mice. (A) The chemical structure of Apelin‐13. (B) Schematic illustration of Apelin‐13 administration and behavioral test time points. (C) Representative H&E staining images at Day 3 after ICH. Scale bar = 500/50 μm. (D) Quantification of brain water content (mean ± SD, *n* = 6). (E) Forelimb placing and (F) Rota‐rod assessment at 0, 1, and 3 days after ICH (mean ± SD, *n* = 6). *p < 0.05, **p < 0.01, ***p < 0.0001.

The chemical structure of Apelin‐13 is presented in Figure 1A. Mice received an intracerebroventricular injection of Apelin‐13 (60 μg/kg) immediately after ICH induction, followed by daily injections (Figure 1B). H&E staining showed substantial hematoma and brain damage in the ICH + Vehicle group, whereas Apelin‐13 treatment significantly attenuated these injuries (Figure 1C). To assess brain edema following ICH, brain water content was assessed. The results indicated a notable elevation in the water content of the ipsilateral cortex and basal ganglia in ICH mice, which was significantly mitigated by Apelin‐13 treatment (Figure 1D). Furthermore, behavioral tests revealed that Apelin‐13 significantly improved neurological function in ICH mice, as evidenced by enhanced forelimb placing scores and rota‐rod performance (Figure 1E,F). Our findings highlighted the therapeutic prospect of Apelin‐13 in the treatment of ICH.

### 3.2 Apelin‐13 Attenuated BBB Dysfunction in ICH Mice

**FIGURE 2:**
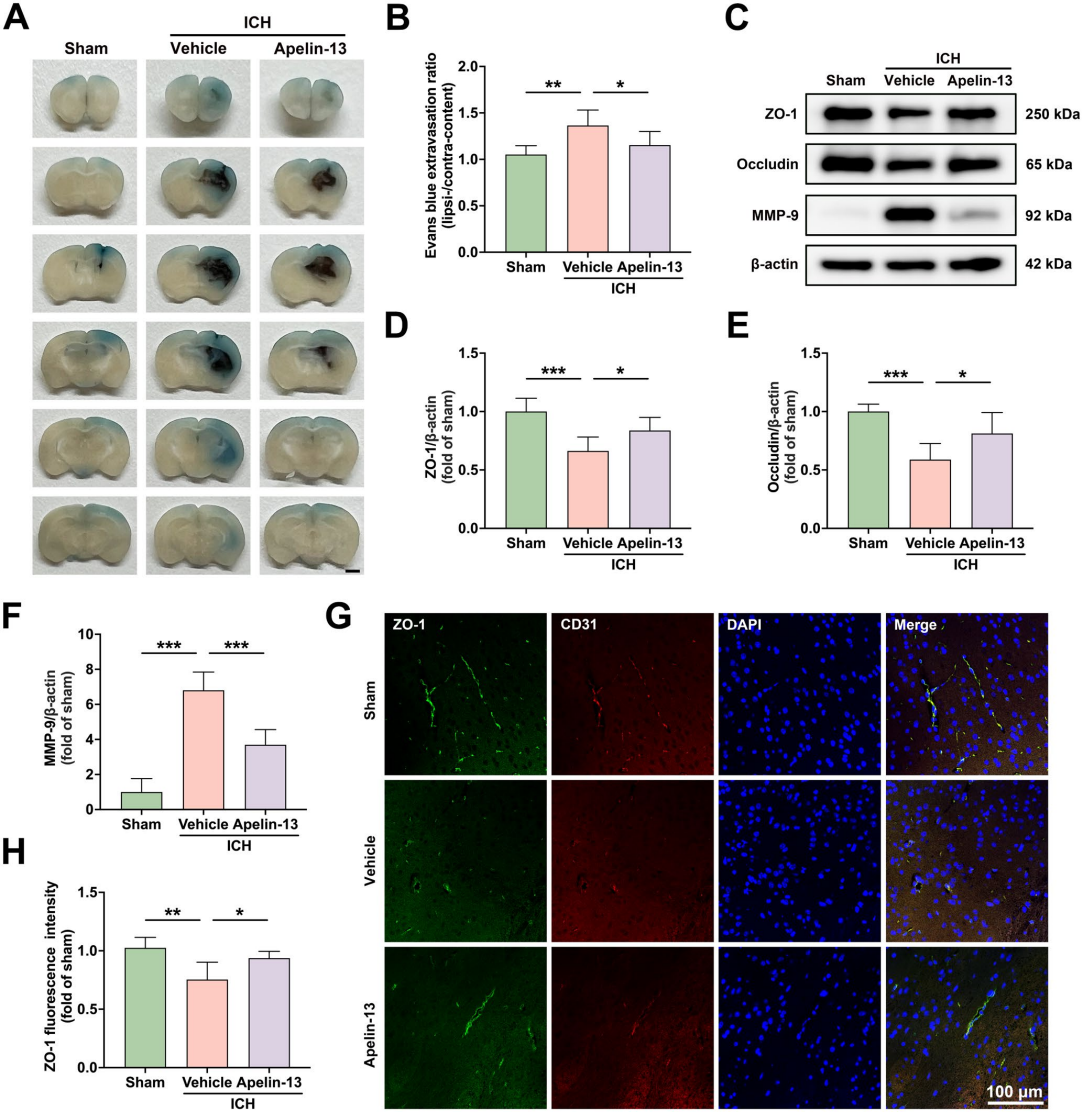
Apelin‐13 attenuated BBB dysfunction following ICH in mice. (A) Representative images of EB extravasation 3 days after ICH, scale bar = 1 mm. (B) Quantification of EB leakage (mean ± SD, *n* = 6). (C) Western blot analysis of ZO‐1, occludin, and MMP‐9 at Day 3 after ICH. (D–F) Quantification of ZO‐1, occludin, and MMP‐9 protein band intensities (mean ± SD, *n* = 6). (G) Double immunofluorescence staining of ZO‐1 and CD31 in perihematomal region. Scale bar = 100 μm. (H) Quantification of ZO‐1 fluorescence intensity (mean ± SD, *n* = 5). *p < 0.05, **p < 0.01, ***p < 0.0001.

To assess BBB permeability, EB extravasation was measured. The results revealed that ICH‐induced BBB compromise was significantly attenuated by Apelin‐13 treatment (Figure 2A,B). To further investigate the protective role of Apelin‐13 in maintaining BBB function following ICH, we conducted western blot and immunofluorescence analyses. Western blotting demonstrated significant downregulation of zonula occludens‐1 (ZO‐1) and occludin, as well as upregulation of MMP‐9 following ICH, which were significantly reversed by Apelin‐13 (Figure 2C–F). Similarly, immunofluorescence analysis indicated a significant elevation of ZO‐1 expression in peri‐hematomal regions following Apelin‐13 administration (Figure 2G,H). These data collectively demonstrated that Apelin‐13 effectively attenuated BBB disruption following ICH in mice.

### 3.3 Apelin‐13 Reduced Hemin‐Induced Apoptosis in bEnd.3 Cells

**FIGURE 3:**
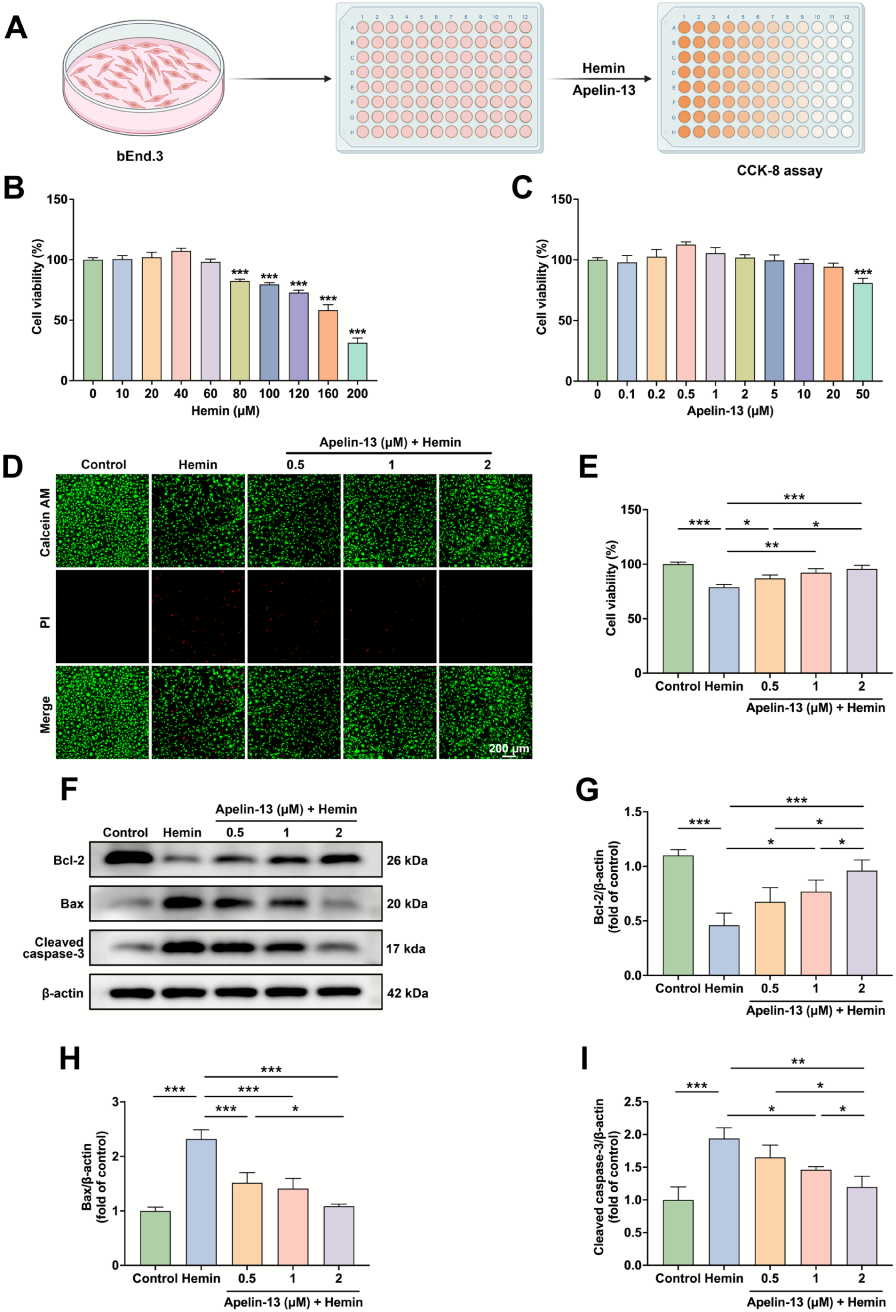
Apelin‐13 inhibited hemin‐induced apoptosis in bEnd.3 cells. (A) Schematic illustration of the cell viability assay. (B, C) The viability of bEnd.3 cells treated with hemin or Apelin‐13 for 24 h was evaluated by CCK‐8 assay (mean ± SD, *n* = 3). (D) Calcein AM/PI staining and (E) CCK‐8 quantification (mean ± SD, *n* = 3) in bEnd.3 cells under different treatments. Scale bar = 200 μm. (F) Western blot analysis of Bcl‐2, Bax, and cleaved caspase‐3 in bEnd.3 cells induced by hemin for 24 h. (G–I) Quantification of Bcl‐2, Bax, and cleaved caspase‐3 protein band intensities (mean ± SD, *n* = 3). *p < 0.05, **p < 0.01, ***p < 0.0001.

In vitro ICH models were induced by exposing bEnd.3 cells to hemin for 24 h. The cytotoxicity of hemin and Apelin‐13 was measured with the CCK‐8 assay (Figure 3A). Our data demonstrated the concentration‐dependent reduction of bEnd.3 cell viability induced by hemin (≥ 80 μM), whereas Apelin‐13 (< 50 μM) exhibited no cytotoxicity (Figure 3B,C). Next, we performed Calcein‐AM/PI staining and CCK‐8 assay to investigate whether Apelin‐13 could protect against hemin‐induced cell death. The experimental data revealed that treatment with 80 μM hemin induced significant cytotoxicity in bEnd.3 cells, which was substantially attenuated by Apelin‐13 (Figure 3D,E). Western blotting demonstrated that hemin induction significantly upregulated pro‐apoptotic proteins Bax and cleaved caspase‐3 and downregulated anti‐apoptotic protein Bcl‐2. Apelin‐13 treatment dose‐dependently reversed these changes (Figure 3F–I). These results revealed that Apelin‐13 reduced hemin‐induced apoptosis in bEnd.3 cells.

### 3.4 Apelin‐13 Mitigated Hemin‐Induced Endothelial Hyperpermeability in bEnd.3 Cells

**FIGURE 4:**
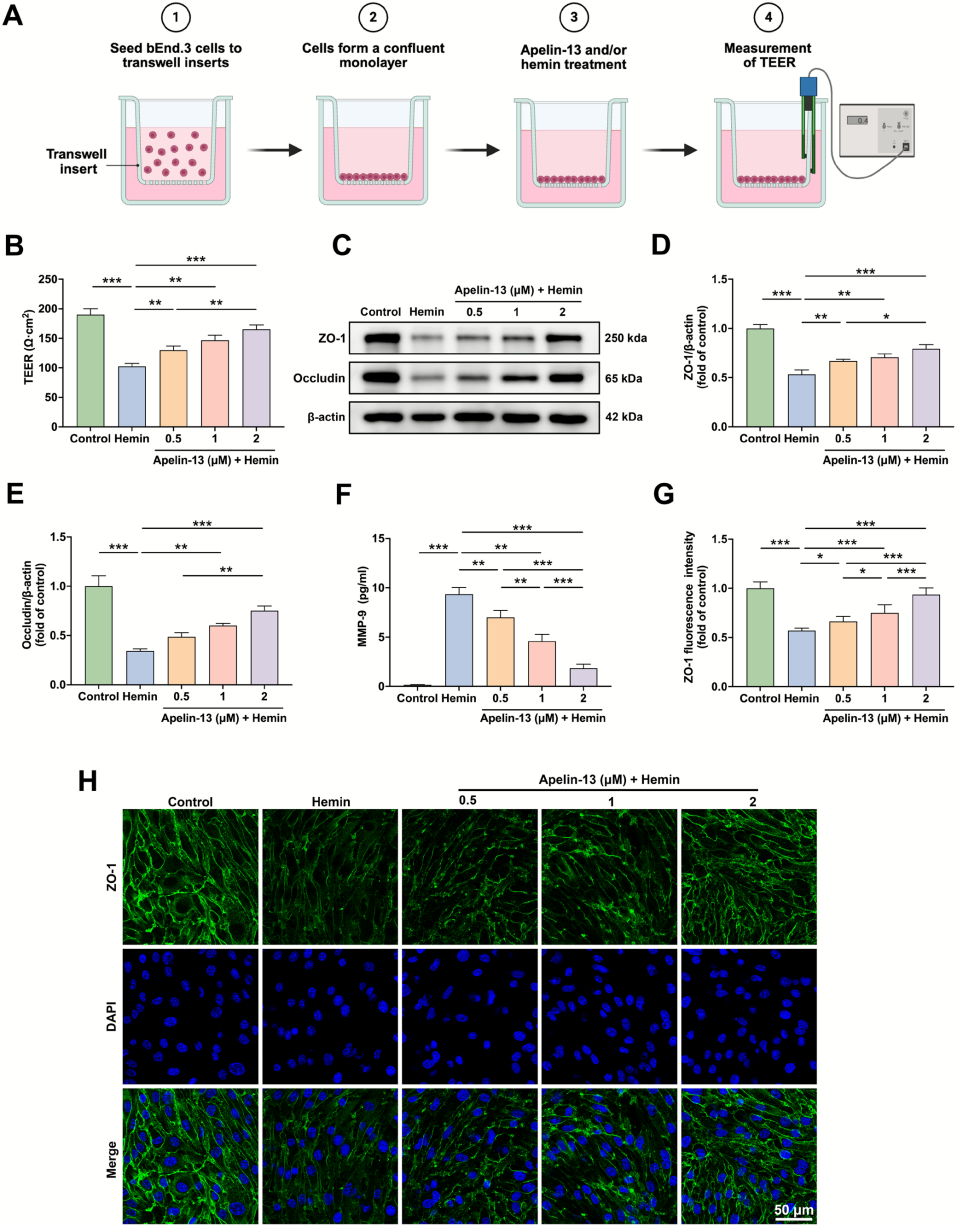
Apelin‐13 mitigated hemin‐induced endothelial hyperpermeability in bEnd.3 cells. (A) Schematic illustration of confluent monolayer establishment and TEER measurement. (B) TEER of the bEnd.3 cell monolayers (mean ± SD, *n* = 3). (C) Western blot analysis of ZO‐1 and occludin in bEnd.3 cells stimulated by hemin for 24 h. (D, E) Quantification of ZO‐1 and occludin protein band intensities (mean ± SD, *n* = 3). (F) MMP‐9 levels in hemin‐induced bEnd.3 cells were measured by ELISA (mean ± SD, *n* = 3). (G) Quantification of ZO‐1 fluorescence intensity (mean ± SD, *n* = 3). (H) Immunofluorescence staining of ZO‐1 in hemin‐induced bEnd.3 cells. Scale bar = 50 μm. *p < 0.05, **p < 0.01, ***p < 0.001.

To establish monolayers, cells were seeded on the transwell filters, which were exposed to hemin (80 μM) for 24 h to simulate in vitro ICH conditions (Figure 4A). Hemin stimulation significantly reduced the TEER of bEnd.3 monolayers, whereas Apelin‐13 treatment effectively ameliorated this damage (Figure 4B). Western blot analysis indicated that hemin stimulation significantly reduced ZO‐1 and occludin levels, which were dose‐dependently restored by Apelin‐13 treatment (Figure 4C–E). ELISA results showed that Apelin‐13 administration significantly counteracted hemin‐induced MMP‐9 upregulation (Figure 4F). Additionally, immunofluorescence analysis further confirmed that the marked reduction of ZO‐1 levels in hemin‐induced bEnd.3 cells was significantly rescued by Apelin‐13 (Figure 4G,H). These findings indicated that hemin‐induced endothelial hyperpermeability in bEnd.3 cells was effectively mitigated by Apelin‐13 treatment.

### 3.5 Apelin‐13 Protected bEnd.3 Cells From Hemin‐Induced Oxidative Stress

**FIGURE 5:**
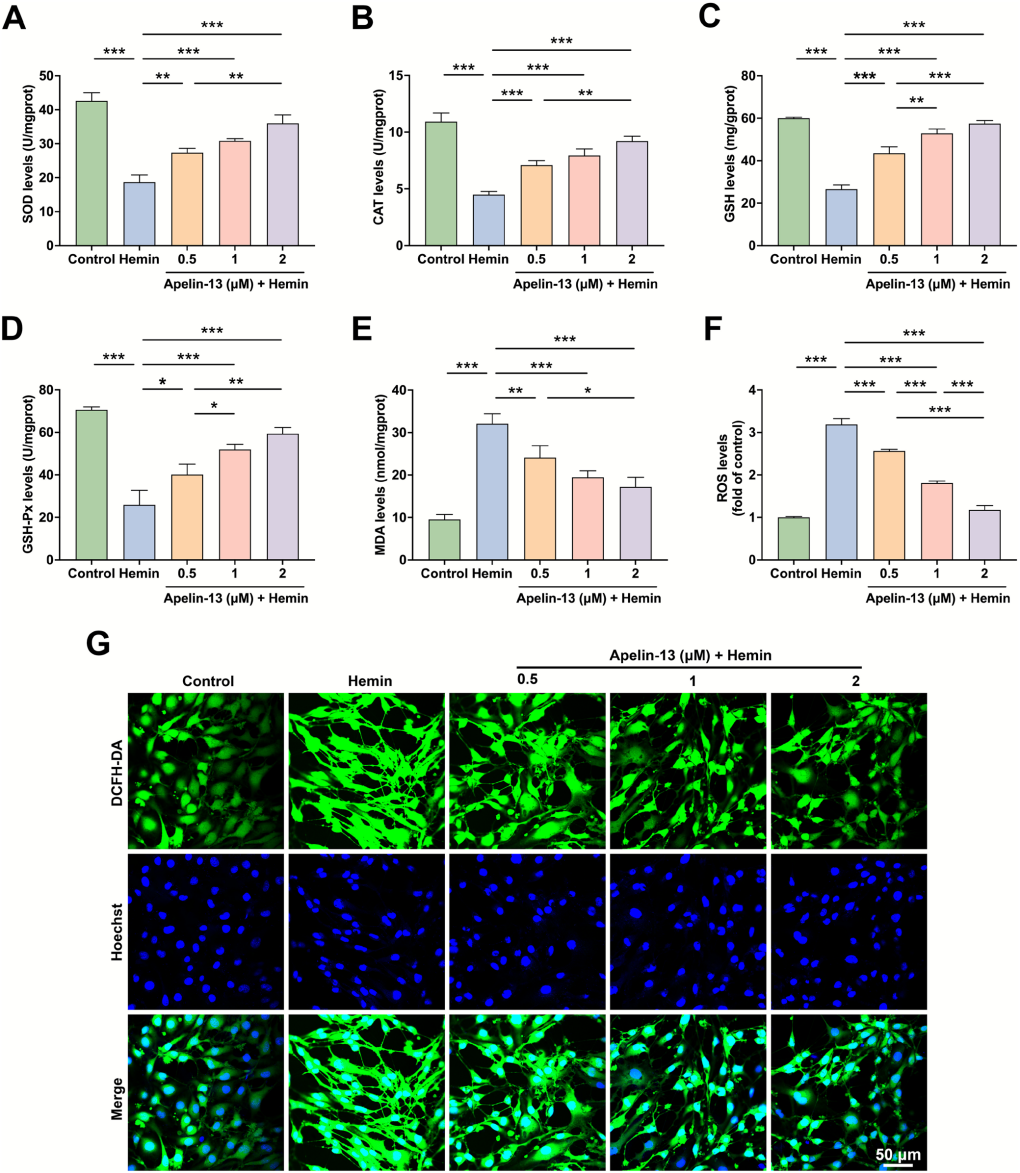
Apelin‐13 protected bEnd.3 cells from hemin‐induced oxidative stress. The levels of SOD (A), CAT (B), GSH (C), GSH‐Px (D), MDA (E), and ROS (F) were determined according to the “Methods” section (mean ± SD, *n* = 3) (G) Representative fluorescence images of DCFH‐DA staining indicating ROS levels in hemin‐induced bEnd.3 cells. Scale bar = 50 μm. *p < 0.05, **p < 0.01, ***p < 0.0001.

We next investigated the antioxidant effects of Apelin‐13 in bEnd.3 cells exposed to hemin. Significant decreases in SOD, CAT, GSH, GSH‐Px levels were observed in hemin‐stimulated bEnd.3 cells compared with the controls. Apelin‐13 treatment dose‐dependently restored these antioxidant protein levels (Figure 5A–D). Meanwhile, hemin stimulation increased MDA and ROS levels, both of which were significantly mitigated by Apelin‐13 (Figure 5E–G). Our findings revealed that Apelin‐13 conferred antioxidant protection in hemin‐induced bEnd.3 cells.

### 3.6 Apelin‐13 Modulated Keap1/Nrf2 Signaling in Hemin‐Induced bEnd.3 Cells

**FIGURE 6:**
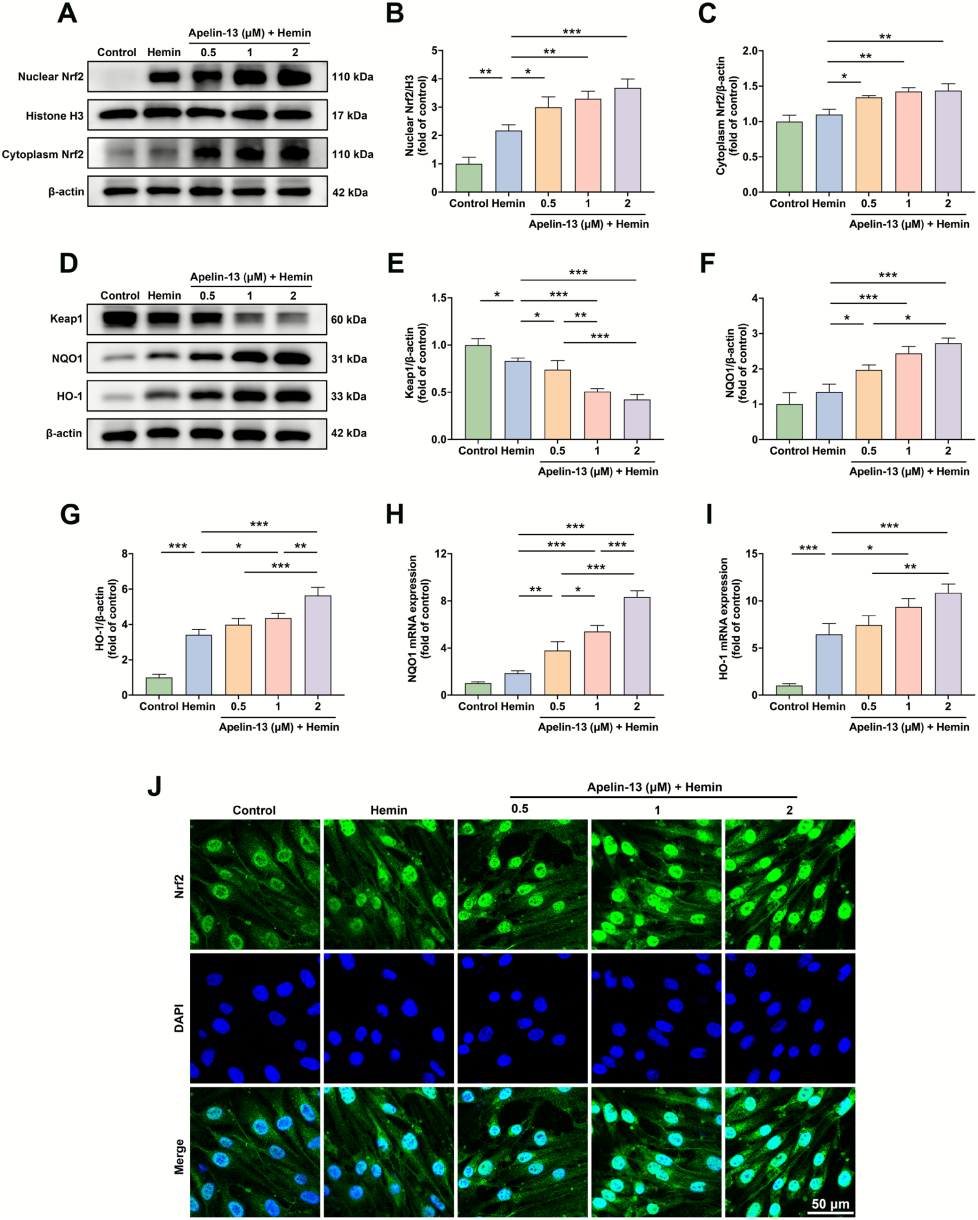
Apelin‐13 activated the Keap1/Nrf2 pathway in hemin‐induced bEnd.3 cells. (A) Western blot analysis of nuclear and cytoplasmic Nrf2 in hemin‐induced bEnd.3 cells. (B, C) Quantification of the nuclear and cytoplasmic Nrf2 protein band intensities (mean ± SD, *n* = 3). (D) Western blot analysis of Keap1, NQO1, and HO‐1 in bEnd.3 cells stimulated by hemin for 24 h. (E–G) Quantification of Keap1, NQO1, and HO‐1 protein band intensities (mean ± SD, *n* = 3). (H, I) The relative mRNA expression levels of NQO1 and HO‐1 were assessed with qRT‐PCR. (J) Immunofluorescence staining of Nrf2 in hemin‐induced bEnd.3 cells. Scale bar = 50 μm. *p < 0.05, **p < 0.01, ***p < 0.0001.

The Keap1/Nrf2 signaling plays a pivotal role in maintaining antioxidant homeostasis following cerebrovascular events [8]. To examine the potential influence of Apelin‐13 on the Keap1/Nrf2 signaling, western blot analysis was performed in hemin‐induced bEnd.3 cells. The results indicated that Apelin‐13 dose‐dependently increased Nrf2 expression in both the cytoplasmic and nuclear compartments compared to the hemin group (Figure 6A–C). Furthermore, Apelin‐13 treatment significantly reduced Keap1, a negative regulator of Nrf2, while concurrently upregulating key Nrf2 downstream targets, comprising NADPH quinone oxidoreductase 1 (NQO1) and heme oxygenase‐1 (HO‐1) (Figure 6D–G). qRT‐PCR analysis further showed that Apelin‐13 significantly upregulated NQO1 and HO‐1 mRNA expression levels (Figure 6H,I). Consistent with these findings, the immunofluorescence analysis indicated that Apelin‐13 significantly facilitated Nrf2 nuclear translocation (Figure 6J). Taken together, these findings demonstrated that Apelin‐13 modulated the Keap1/Nrf2 pathway in hemin‐induced bEnd.3 cells, enhancing Nrf2 nuclear translocation and transcriptional activity. To further delineate the contribution of APJ to the activation of the Keap1/Nrf2 pathway by Apelin‐13, we employed APJ‐specific shRNA in hemin‐stimulated bEnd.3 cells, using a scrambled shRNA plasmid as a negative control. The efficacy of APJ silencing was confirmed by western blot analysis (Figure S1A,B). Moreover, APJ knockdown partially abolished the effects of Apelin‐13 on the Keap1/Nrf2 pathway, as evidenced by reduced Nrf2 levels in both nuclear and cytoplasmic fractions (Figure S2C–E). Consistent with this finding, APJ silencing also reversed the Apelin‐13‐mediated downregulation of Keap1 and upregulation of its downstream targets, NQO1 and HO‐1 (Figure S1F–I). Our findings confirmed that APJ knockdown significantly blocked the effects of Apelin‐13 on the Keap1/Nrf2 pathway, demonstrating that APJ is a necessary mediator.

### 3.7 Apelin‐13 Alleviated Hemin‐Induced BBB Dysfunction In Vitro by Targeting the Keap1/Nrf2 Signaling

**FIGURE 7:**
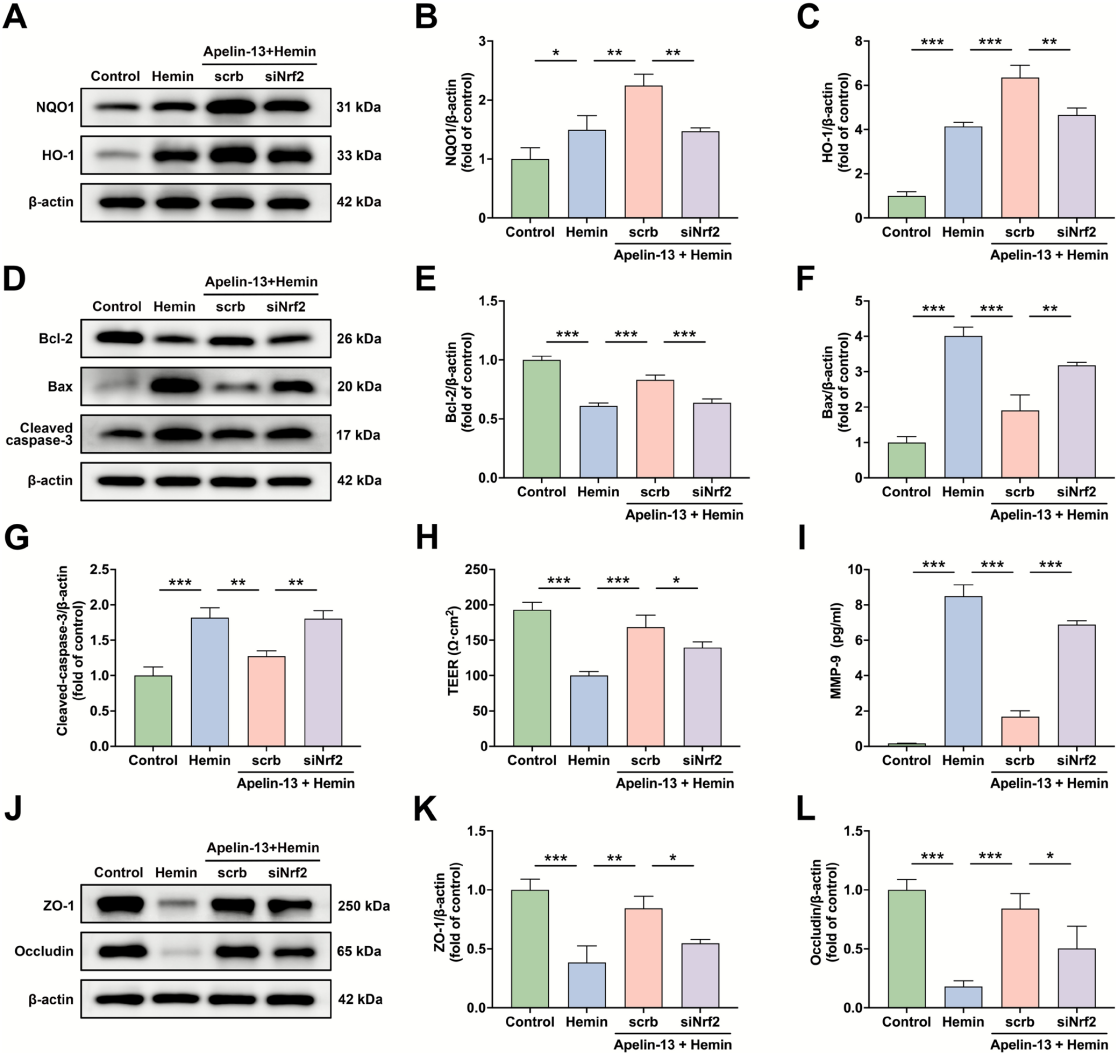
Apelin‐13 alleviated hemin‐induced BBB dysfunction in vitro via targeting the Keap1/Nrf2 signaling. (A) bEnd.3 cells were pretreated with Nrf2‐specific shRNA or scrambled shRNA for 48 h, then incubated with Apelin‐13 and/or hemin, followed by NQO1 and HO‐1 western blot analysis. (B, C) Quantification of NQO1 and HO‐1 protein band intensities (mean ± SD, *n* = 3). (D) Effects of siNrf2 on Bcl‐2, Bax, and cleaved caspase‐3 expression were evaluated by western blot analysis. (E–G) Quantification of Bcl‐2, Bax, and cleaved caspase‐3 protein band intensities (mean ± SD, *n* = 3). (H) Effects of siNrf2 on bEnd.3 cell monolayer permeability (mean ± SD, *n* = 3). (I) Effects of siNrf2 on MMP‐9 secretion (mean ± SD, *n* = 3). (J) Effects of siNrf2 on ZO‐1 and occludin expression were determined using western blot analysis. (K, L) Quantification of ZO‐1 and occludin protein band intensities (mean ± SD, *n* = 3). *p < 0.05, **p < 0.01, ***p < 0.0001.

To investigate the involvement of the Keap1/Nrf2 signaling in Apelin‐13‐mediated BBB protection, we silenced Nrf2 expression using shRNA in hemin‐induced bEnd.3 cells. A control shRNA plasmid (scrambled sequence) served as a negative control. Given that Apelin‐13 exhibited optimal neuroprotective effects at a concentration of 2 μM, this concentration was employed in our subsequent experiments. Western blot analysis confirmed successful Nrf2 knockdown, evidenced by significantly reduced Nrf2 protein levels (Figure S2A,B). Subsequent experiments demonstrated that Nrf2 knockdown blocked the upregulation of NQO1 and HO‐1 mediated by Apelin‐13 in hemin‐stimulated bEnd.3 cells (Figure 7A–C). Furthermore, Nrf2 silencing suppressed the ability of Apelin‐13 to downregulate ROS levels in experimental models (Figure S2C). Notably, Nrf2 silencing also abrogated the anti‐apoptotic effects of Apelin‐13 in hemin‐stimulated bEnd.3 cells, specifically by reversing its regulatory actions on apoptotic markers (Figure 7D–G) and cell viability (Figure S2D). Finally, Nrf2 knockdown eliminated the protective effects of Apelin‐13 against barrier dysfunction, evidenced by decreased TEER (Figure 7H), elevated MMP‐9 levels (Figure 7I), and reduction of ZO‐1 and occludin (Figure 7J–L). These findings collectively indicated that Apelin‐13 mitigated hemin‐induced BBB dysfunction through modulating the Keap1/Nrf2 signaling.

## 4 Discussion

In this study, ICH was induced in mice with collagenase, Apelin‐13 (60 μg/kg) was injected intracerebroventricularly immediately post‐ICH followed by daily administration. We identified the neuroprotective role of Apelin‐13 in ICH mice that mitigated brain damage, reduced brain edema, and improved neurological function. BBB disruption, characterized by endothelial tight junction damage, is a critical contributor to perihematomal brain edema [5]. Beyond the mechanical injury, MMP‐9 serves as an important mediator of BBB breakdown following ICH by facilitating the proteolytic degradation of tight junction proteins (e.g., ZO‐1, claudins, and occludin) and extracellular matrix [31]. Following ICH, MMP‐9 undergoes rapid upregulation in both expression and activity, aggravating BBB compromise and brain edema [32]. Our findings revealed that Apelin‐13 significantly attenuated ICH‐induced BBB disruption, indicated by lower EB extravasation, reduced MMP‐9 levels, and increased ZO‐1 and occludin expression. Next, we conducted in vitro experiments to investigate the molecular mechanisms through which Apelin‐13 attenuated BBB disruption following ICH.

Considering the well‐established contribution of oxidative damage‐induced endothelial apoptosis to BBB dysfunction after brain injury [33, 34], the bEnd.3 mouse brain microvascular endothelial cells were employed in our subsequent experiments. Cells were stimulated with 80 μM hemin to simulate ICH conditions in vitro. We observed that Apelin‐13 dose‐dependently attenuated hemin‐induced cytotoxicity in bEnd.3 cells. Additionally, hemin exposure markedly suppressed Bcl‐2 expression and enhanced Bax and cleaved caspase‐3 expression. These changes were significantly abolished by Apelin‐13. Bcl‐2 and Bax belong to the Bcl‐2 protein family but exert opposing effects in apoptosis regulation: Bcl‐2 inhibits apoptosis while Bax facilitates it [35, 36]. Caspase‐3, an effector cysteine protease, undergoes proteolytic cleavage and activation by initiator caspases (e.g., caspase‐8 or caspase‐9), ultimately triggering the irreversible execution of apoptotic cell death [37]. The present findings demonstrated that Apelin‐13 significantly reduced hemin‐induced apoptosis in bEnd.3 cells, aligning with previous evidence that highlights the anti‐apoptotic capacity of Apelin‐13 across various tissues [38, 39, 40].

To investigate Apelin‐13's neuroprotective effects against BBB dysfunction in vitro, we established bEnd.3 monolayers on transwell inserts to model the endothelial barrier. TEER was measured to evaluate paracellular permeability. Our data revealed that the hemin‐induced hyperpermeability in bEnd.3 monolayers was significantly restored by Apelin‐13 treatment, suggesting its protective role in preserving barrier function. Subsequent experimental results demonstrated that the significant downregulation of tight junction proteins and upregulation of MMP‐9 induced by hemin were both reversed by Apelin‐13 treatment. These in vitro findings corroborated our previous in vivo observations, providing accumulating evidence that Apelin‐13 protected against BBB disruption after ICH.

Following ICH, erythrocyte lysis products are critical contributors to secondary brain injury and perihematomal edema. Iron overload, primarily derived from hemoglobin degradation, triggers excessive ROS/RNS generation and subsequent oxidative damage to DNA, lipids, and proteins, thus triggering cell apoptosis [41]. The primary antioxidant defense system counters this oxidative assault through enzymatic (SOD, CAT, and GSH‐Px) and non‐enzymatic (GSH) components that maintain redox homeostasis [42]. Previous studies demonstrated that Apelin‐13 played antioxidant roles in many tissues. In osteoporosis models, Apelin‐13 effectively ameliorated oxidative stress and enhanced osteogenic function [43]. In adipose tissue, Apelin‐13 significantly attenuated uric acid‐induced oxidative stress [44]. Another study conducted in asthma models revealed that Apelin‐13 protected against allergen‐induced airway oxidative damage [45]. In this study, hemin exposure significantly suppressed endogenous antioxidant defenses, evidenced by the downregulation of antioxidant enzymes. Apelin‐13 dose‐dependently restored these critical redox regulators. Meanwhile, Apelin‐13 treatment substantially downregulated oxidative stress markers, reducing MDA (a lipid peroxidation indicator) levels and suppressing ROS generation. These findings indicated that the neuroprotective properties of Apelin‐13 might be attributed to its antioxidant capabilities.

**FIGURE 8:**
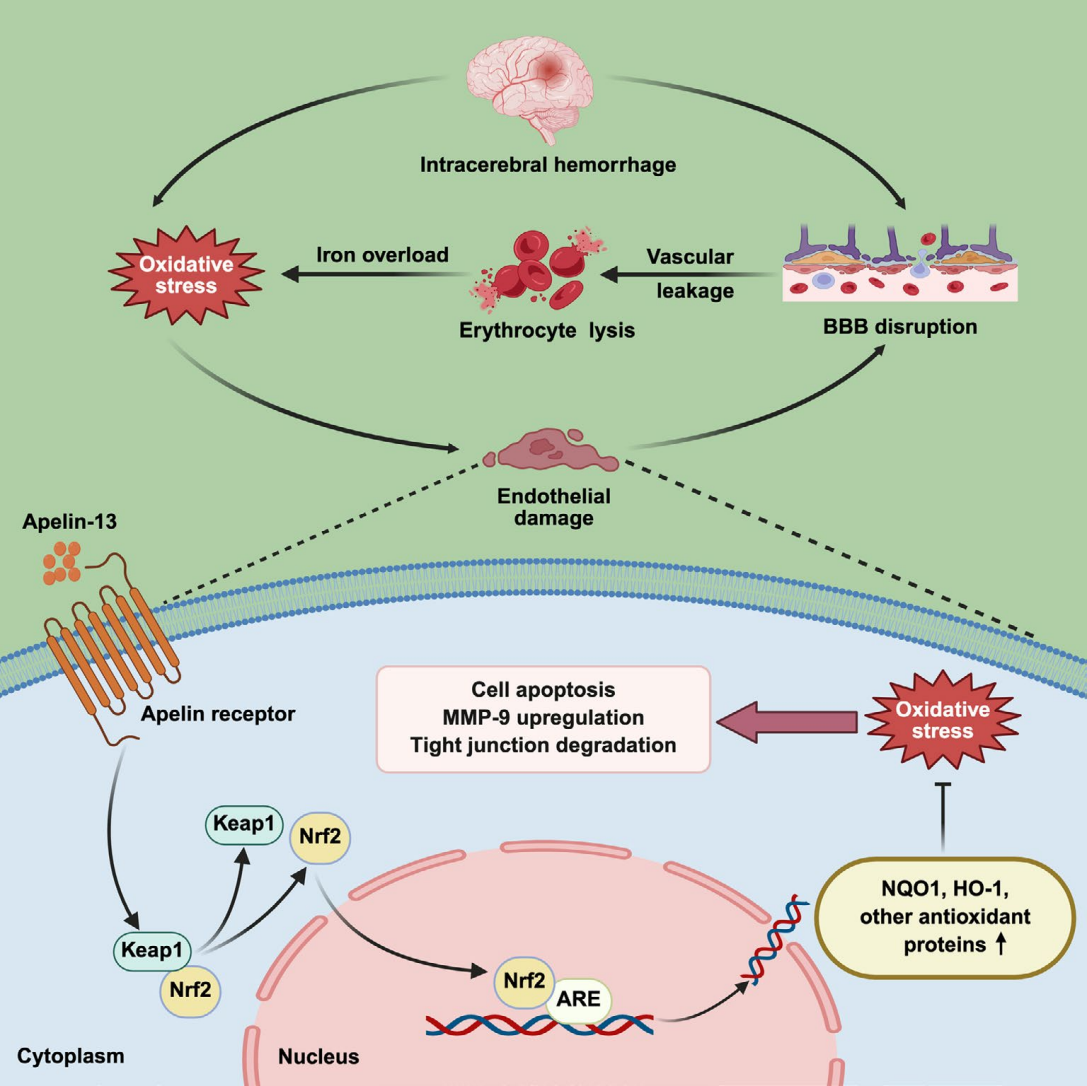
Potential mechanisms underlying the neuroprotective role of Apelin‐13 in ICH. Apelin‐13 attenuates ICH‐induced BBB disruption through activating the Keap1/Nrf2 signaling.

Nrf2, encoded by the *NFE2L2* gene, serves as a critical transcription regulator of endogenous antioxidant defenses [46]. Under unstressed conditions, Nrf2 is constitutively bound and sequestered in the cytoplasm by Keap1. More specifically, Keap1 binds to Nrf2 and bridges it to the Cul3/RBX1 ubiquitin ligase complex, thereby facilitating Nrf2 ubiquitination and its subsequent proteasomal degradation [47]. Under oxidative stress, the specific cysteine residues of Keap1 undergo modification, contributing to Nrf2 liberation and nuclear translocation [48]. The nuclear‐localized Nrf2 then binds to antioxidant response elements (AREs), thereby enhancing the transcription of cytoprotective genes associated with the regulation of oxidative stress, apoptosis, ferroptosis, inflammation, and autophagy [49]. As critical Nrf2‐ARE effector proteins, NQO1 and HO‐1 execute cytoprotection primarily via antioxidant mechanisms [50]. Accumulating evidence indicates that Nrf2 signaling activation contributes to Apelin‐13‐mediated neuroprotection [25, 26]. In this study, Apelin‐13 treatment significantly enhanced Nrf2 nuclear accumulation and upregulated NQO1 and HO‐1 levels compared to the hemin group. Correspondingly, Apelin‐13 administration markedly decreased Keap1 expression. These findings collectively indicated that the cytoprotective effects of Apelin‐13 were largely mediated through the Keap1/Nrf2 signaling. Furthermore, APJ knockdown using a specific shRNA largely abrogated the regulatory effects of Apelin‐13 on this pathway, establishing the necessity of this specific ligand‐receptor interaction for pathway regulation. To further validate the downstream mechanisms, Nrf2‐targeting shRNA was used to silence Nrf2. Our experimental data revealed that Nrf2 silencing abolished the cytoprotective effects of Apelin‐13 in hemin‐induced bEnd.3 cells. Specifically, Nrf2 knockdown significantly eliminated the protective actions of Apelin‐13 against hemin‐induced damage, including the preservation of BBB integrity, suppression of apoptosis, and attenuation of oxidative injury. These findings provided compelling in vitro evidence that Apelin‐13, by binding to APJ, activated the Keap1/Nrf2 signaling pathway, thereby ameliorating ICH‐induced BBB dysfunction and brain edema (Figure 8).

## 5 Conclusion

To conclude, our study showed that Apelin‐13 alleviated ICH‐induced BBB disruption both in vivo and in vitro. These neuroprotective effects are partially mediated by engaging the APJ receptor and activating the endothelial Keap1/Nrf2 signaling pathway. Our findings underscore the therapeutic potential of Apelin‐13 in preserving BBB integrity following ICH through enhanced antioxidant defenses.

## Author Contributions

P.G. performed the experiments, conducted the statistical analysis, and drafted the manuscript. R.S. performed the experimental design and statistical analysis. J.Q. and L.Q. performed behavioral assays. V.W.Y. and M.X. conceived the experiments, revised the manuscript, and acquired funding.

## Funding

This work was supported by the National Natural Science Foundation of China, U25A2065, W2541024.

## Ethics Statement

All experimental procedures were approved by the Ethics Committee of Zhengzhou University (No. KY2024231).

## Conflicts of Interest

The authors declare no conflicts of interest.

## Supporting information


**Figure S1:** APJ knockdown abrogated the effects of Apelin‐13 on the Keap1/Nrf2 signaling pathway in hemin‐stimulated bEnd.3 cells. (A, B) Effective silencing of APJ by its specific shRNA was confirmed by western blot analysis (mean ± SD, *n* = 3). (C) Effects of siAPJ on the expression levels of nuclear and cytoplasmic Nrf2. (D, E) Quantification of nuclear and cytoplasmic Nrf2 protein band intensities (mean ± SD, *n* = 3). (F) Effects of siAPJ on the expression of Keap1, NQO1, and HO‐1. (G–I) Quantification of Keap1, NQO1, and HO‐1 protein band intensities (mean ± SD, *n* = 3). *p < 0.05, **p < 0.01, ***p < 0.0001.

**Figure S2:** Nrf2 silencing abolished the protective effects of Apelin‐13 against hemin‐induced oxidative stress and cell death in bEnd.3 cells. (A, B) Western blot analysis confirmed the effective silencing of Nrf2 using its specific shRNA (mean ± SD, *n* = 3). (C) Effects of siNrf2 on ROS levels (mean ± SD, *n* = 3). (D) Effects of siNrf2 on bEnd.3 cell viability (mean ± SD, *n* = 3). *p < 0.05, **p < 0.01, ***p < 0.0001.

## Data Availability

The data that support the findings of this study are available from the corresponding author upon reasonable request.
