# Supplementary figures and images for: Apelin‐13 Attenuates Blood–Brain Barrier Dysfunction Following Intracerebral Hemorrhage via Targeting the Keap1/Nrf2 Signaling
Source: CNS Neurosci Ther. 2025 Dec 29;31(12):e70706. doi: 10.1002/cns.70706 (PMC12746050; doi:10.1002/cns.70706)

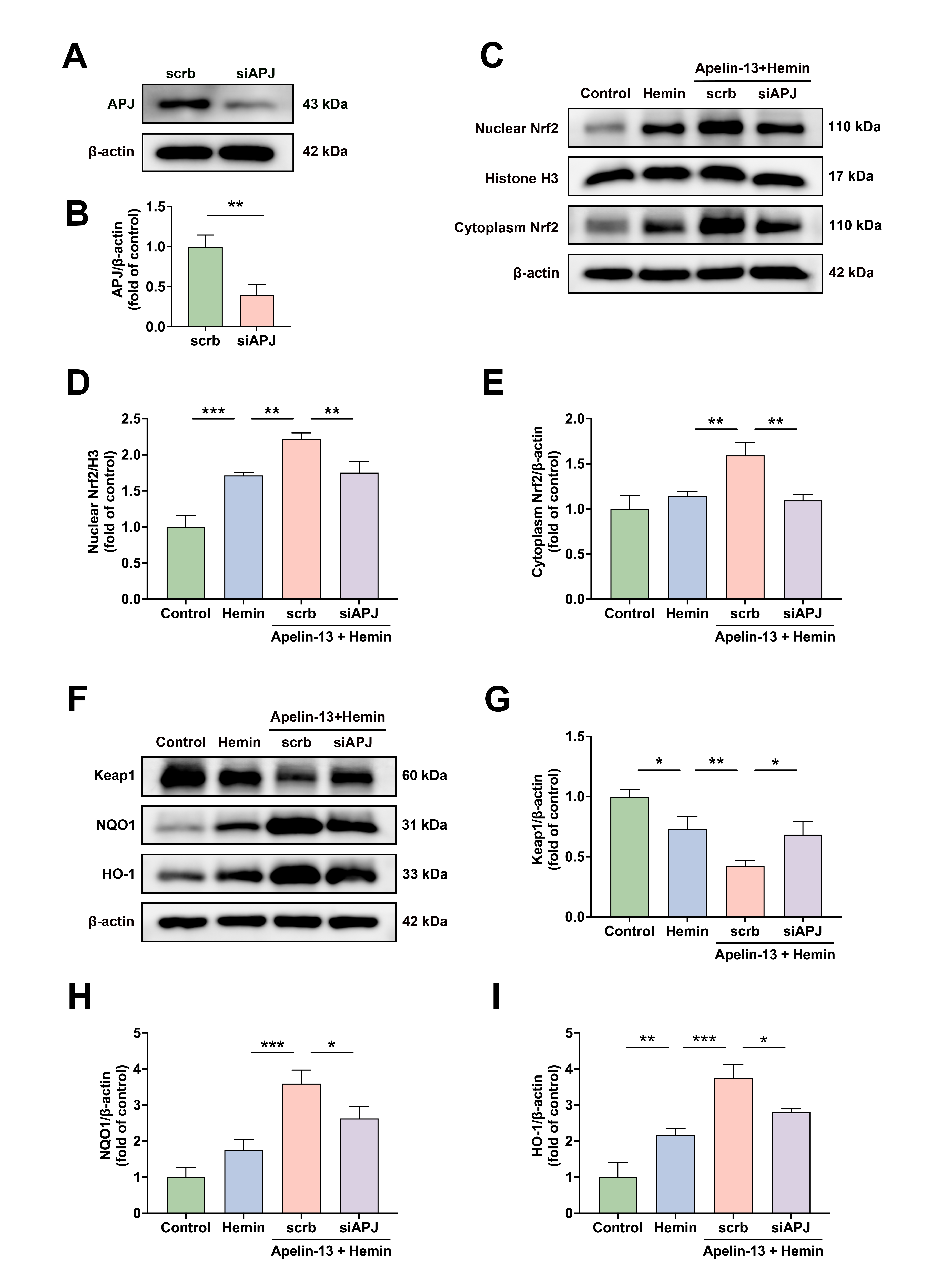

Supplement: Supplementary file 1 — Figure S1: APJ knockdown abrogated the effects of Apelin‐13 on the Keap1/Nrf2 signaling pathway in hemin‐stimulated bEnd.3 cells. (A, B) Effective silencing of APJ by its specific shRNA was confirmed by western blot analysis (mean ± SD, n = 3). (C) Effects of siAPJ on the expression levels of nuclear and cytoplasmic Nrf2. (D, E) Quantification of nuclear and cytoplasmic Nrf2 protein band intensities (mean ± SD, n = 3). (F) Effects of siAPJ on the expression of Keap1, NQO1, and HO‐1. (G–I) Quantification of Keap1, NQO1, and HO‐1 protein band intensities (mean ± SD, n = 3). *p < 0.05, **p < 0.01, ***p < 0.0001. [file CNS-31-e70706-s001.png]

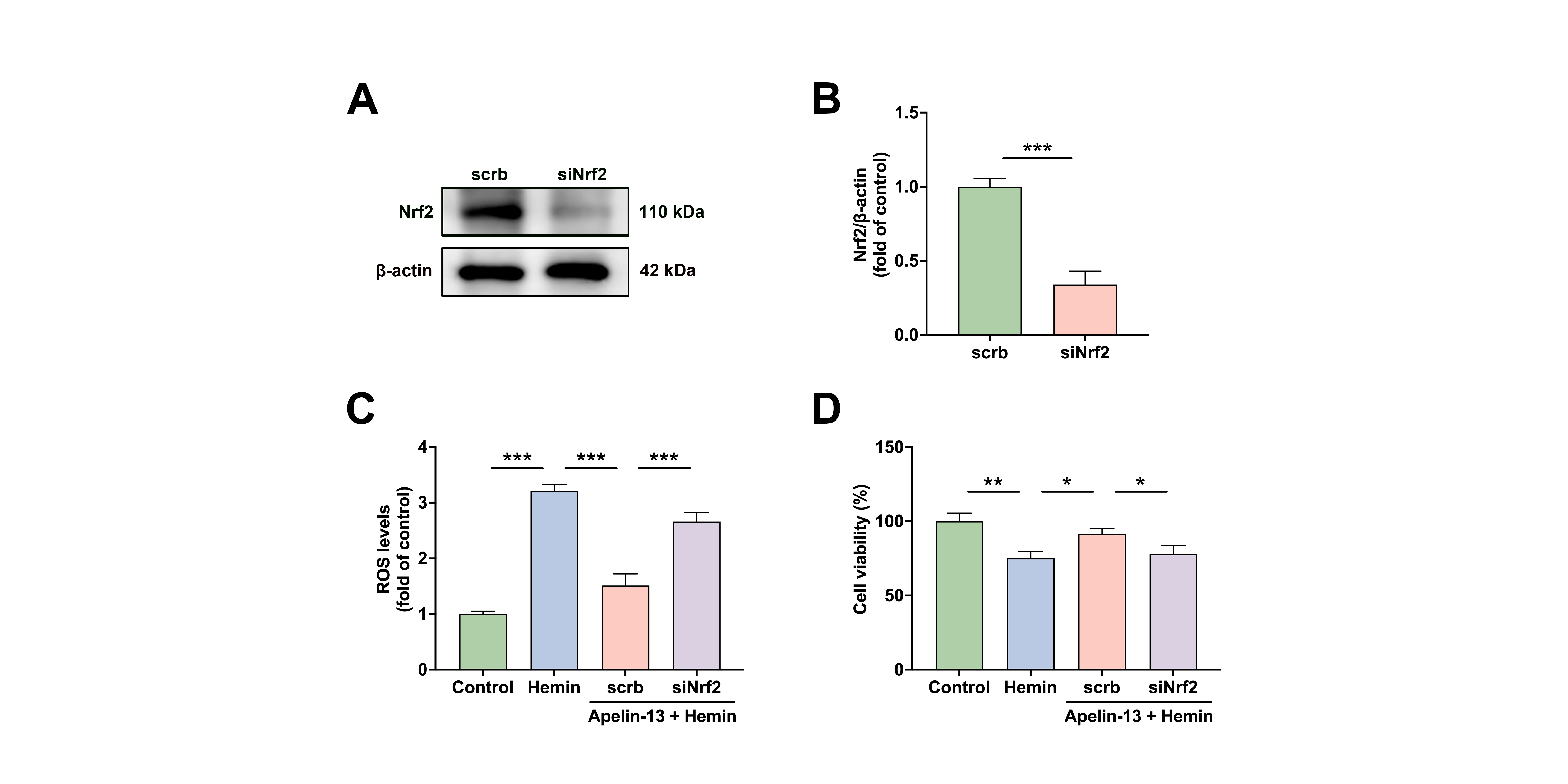

Supplement: Supplementary file 2 — Figure S2: Nrf2 silencing abolished the protective effects of Apelin‐13 against hemin‐induced oxidative stress and cell death in bEnd.3 cells. (A, B) Western blot analysis confirmed the effective silencing of Nrf2 using its specific shRNA (mean ± SD, n = 3). (C) Effects of siNrf2 on ROS levels (mean ± SD, n = 3). (D) Effects of siNrf2 on bEnd.3 cell viability (mean ± SD, n = 3). *p < 0.05, **p < 0.01, ***p < 0.0001. [file CNS-31-e70706-s002.png]
